# *In vivo* Antiphytoviral Activity of Essential Oils and Hydrosols From *Origanum vulgare*, *Thymus vulgaris*, and *Rosmarinus officinalis* to Control Zucchini Yellow Mosaic Virus and Tomato Leaf Curl New Delhi Virus in *Cucurbita pepo* L.

**DOI:** 10.3389/fmicb.2022.840893

**Published:** 2022-04-25

**Authors:** Anna Taglienti, Livia Donati, Luca Ferretti, Laura Tomassoli, Filippo Sapienza, Manuela Sabatino, Gaia Di Massimo, Simona Fiorentino, Valerio Vecchiarelli, Paolo Nota, Rino Ragno

**Affiliations:** ^1^Research Centre for Plant Protection and Certification, Council for Agricultural Research and Economics, Rome, Italy; ^2^Department of Drug Chemistry and Technology, University “La Sapienza,” Rome, Italy; ^3^Centro Appenninico del Terminillo “Carlo Jucci,” Perugia University, Rieti, Italy

**Keywords:** essential oil, hydrosol, plant virus, antiphytoviral, defense response

## Abstract

In the last decades, the interest in biological activity of natural compounds has been growing. In plant protection, essential oils have been reported to exhibit antiviral, antimycotic, and antiparasitic activities, and are regarded as promising for the formulation of safe antimicrobial agents. Attention has also been focused on hydrosols, the by-products of hydro-distillation of essential oils. Their production is easy, fast, and cheap, and they seem to arise less concern for human health than essential oils. Plant viruses represent a major concern for agricultural crops since no treatment compound is available for virus control. This work was aimed at evaluating the antiphytoviral effectiveness of treatments with three essential oils and corresponding hydrosols extracted from *Origanum vulgare*, *Thymus vulgaris*, and *Rosmarinus officinalis* on *Cucurbita pepo* plants infected by zucchini yellow mosaic virus or tomato leaf curl New Delhi virus. Treatments were applied either concurrently or after virus inoculation to ascertain an inhibition or curative activity, respectively. Symptoms were observed and samplings were performed weekly. Virus titer and expression levels of phenylalanine ammonia lyase gene (PAL) were measured on treated and untreated infected plants by real-time PCR. PAL gene plays an important role in plant defense response as it is involved in tolerance/resistance to phytopathogens. Results indicated that treatments were effective against tomato leaf curl New Delhi virus whether applied simultaneously with the inoculation or after. A major inhibition was observed with *O. vulgare* essential oil and hydrosol, resulting in 10^–4^-fold decrease of virus titer 3 weeks after treatment. Curative activity gave maximum results with all three essential oils and *T. vulgaris* and *R. officinalis* hydrosols, recording from 10^–2^-fold decrease to virus not detected 4 weeks after treatment. An induction of PAL gene expression was recorded at 12 d.p.i. and then was restored to the levels of untreated control. This allows to hypothesize an early plant defense response to virus infection, possibly boosted by treatments. Plant extracts’ composition was characterized by gas chromatography-mass spectrometry. Phenols were largely main components of *O. vulgare* and *T. vulgaris* extracts (carvacrol and thymol, respectively), while extracts from *R. officinalis* were based on monoterpene hydrocarbons (essential oil) and oxygenated monoterpenes (hydrosol).

## Introduction

Essential oils (EOs) are complex mixtures of low molecular weight and volatile compounds that are formed and stored in aromatic plant families (e.g., *Apiaceae*, *Asteraceae*, *Lamiaceae*, *Myrataceae*) particularly rich in these secondary metabolites (Baser and Buchbauer, 2015). They are usually obtained by steam or hydro-distillation (Speranza and Corbo, 2010; Garzoli et al., 2015; Božović et al., 2017), with the components belonging to a wide variety of chemical classes: aliphatic and aromatic compounds, hydrocarbons, alcohols, aldehydes, ketones, esters, phenols, and acids. Many of these substances are reported to display antimicrobial, antiviral, antimycotic, antiparasitic, and insecticidal properties both against mammalian and plant pathogens (Bakkali et al., 2008; Civitelli et al., 2014; Garzoli et al., 2018; Sabatino et al., 2020). In literature, the separated condensed water, saturated of water-soluble essential oil components, produced during the distillation process of EOs is reported under various terms, including hydrosol (HS), hydrolate, hydroflorate, and aromatic water (Rajeswara Rao, 2013). Herein, this extract is referred to as HS. Regardless of the used name, their composition is known to be represented by a small quantity of EO (i.e., < 1 g/L) and by polar, oxygenated, and hydrophilic oil components forming hydrogen bonds with water (Labadie et al., 2016).

Virus infection in plants is a major concern for agricultural crops, causing considerable economical losses worldwide (Lecoq and Katis, 2014). Since no curative treatment consisting of application of compounds of natural or synthetic origin is available for virus control in plants, such as for bacteria and fungi (Rubio et al., 2020), most of defense strategies lie on prevention (e.g., use of sanitarily certified propagation material, control of insect vectors) (Fereres and Raccah, 2015; Golino et al., 2017). Other control measures require the use of resistant varieties developed by breeding (Paris and Brown, 2005; Martín-Hernández and Picó, 2020). Engineered mild virus strains for accomplishing cross-protection or transgenic plants represent promising approaches which, to date, are limited to a few plant/virus species (You et al., 2005; Jia et al., 2017; Hamim et al., 2018). Their employment is further restrained because applied research and use of transgenic plants are forbidden in many countries. When no other measure is feasible, *in vitro* virus elimination is accomplished with different techniques (thermotherapy, meristem culture, cryotherapy, micro-grafting) which are all very expensive, time-consuming, and do not suite all plant/virus pathosystems (Naik and Buckseth, 2018).

Various substances of natural and synthetic origin have been used for plant virus infection control, and in some studies, the antiphytoviral activity of EOs has been reported (Zhao et al., 2017; Raveau et al., 2020). Such treatments aim at moderating symptom expression and limiting the yield losses due to infection by reducing the viral titer or inactivating some viral functional genes. Further, the application of treatments based on natural products is also gaining interest in view of the envisaged restrictions to the synthetic chemicals’ overuse that has been proved to cause environmental and human health issues. Such limitations (e.g., Regulation 1107/2009/EC, 2011) drive the demand for alternative control methods and integrated pest management systems (Lamichhane et al., 2016). Therefore, many studies have focused on the use of natural products for virus control in plants. Trials involving tobacco mosaic virus (TMV) were based on the count of local lesions. Bishop (1995) and Lu (2013) both worked on a *Nicotiana glutinosa* experimental host, reporting an inhibition of local symptoms development upon treatments with EOs from *Melaleuca alternifolia*, ginger, lemon, tea tree, tangerine peel, artemisia, and lemongrass. Dunkić et al. (2010) used *Satureja montana* EO as a treatment for *Chenopodium quinoa* and *Chenopodium amaranticolor* hosts infected with TMV and cucumber mosaic virus (CMV). Further experiments involving *C. quinoa*-CMV pathosystem (Bezić et al., 2011; Vuko et al., 2019) reported the ability of EOs from *Micromeria croatia* and *Teucrium* species to reduce the local lesion number. The effectiveness of *Nigella sativa* seed extract against zucchini yellow mosaic virus (ZYMV) in *C. pepo* was investigated both *in vitro* and *in vivo* (Abdel-Shafi, 2013). Local lesion number and physiological parameters (leaves number, shoot length, etc.) were measured for assessing antiphytoviral activity. The detailed mechanism of such activity is yet to be fully explored. It has been hypothesized that EO components could either directly inactivate viral particles or induce resistance/tolerance response in the host (Othman and Shoman, 2004). HSs have often been considered a waste by-product of EO distillation, but recently they have gained a growing interest due to their reported antimicrobial activity both *in vitro* and *in situ* (Saǧdιç, 2003; Tornuk et al., 2011; Ozturk et al., 2016). Furthermore, their use has been endorsed for their low production cost. In the field of plant protection, antiphytoviral activity of HSs from various plant species against TMV has been reported (Nazlić et al., 2021; Vuko et al., 2021a,b).

Based on these findings, we hypothesized that EOs and HSs could have a potential antiphytoviral activity *in vivo*. In particular, in this study, we aimed at verifying whether EOs and HSs from *Origanum vulgare* (OV), *Thymus vulgaris* (TV), and *Rosmarinum officinalis* (RO) could be effective for the control of ZYMV and tomato leaf curl New Delhi virus (ToLCNDV) in *Cucurbita pepo* L. plants *in vivo*. ZYMV, a member of the genus *Potyvirus*, family *Potyviridae*, is a ssRNA (+) virus efficiently transmitted by aphids in a non-persistent manner. It affects all members of the *Cucurbitaceae* family, including pumpkin, squash, vegetable marrow, zucchini, melon, watermelon, cucumber, and gherkin (Gal-On, 2007). It induces symptoms such as severe leaf mosaic and yellowing. The fruits are stunted, twisted and deformed by raised protuberances, which makes them unmarketable. In cultivated crops, plants cease marketable production within 1–2 weeks post infection. Hence, serious economic losses, reducing yield up to 90%, can occur, particularly in zucchini and marrow crops (Walkey et al., 1992). ToLCNDV is a bipartite begomovirus (family *Geminiviridae*) that was identified for the first time in 1995 in Asia (Padidam et al., 1995), from where it recently spread into the Mediterranean basin (Navas-Castillo et al., 2011; Juárez et al., 2014; Luigi et al., 2016; Bertin et al., 2018), becoming a serious threat to a number of economically important crops and is therefore responsible for severe production losses. ToLCNDV genome is constituted of two circular ssDNA components, named DNA-A and DNA-B. In zucchini plants, it causes a wide and severe symptomatology, including leaf curling, swelling of veins and yellow mosaic on leaves, stems with shortened internodes, and fruits showing skin roughness and reduced size (Panno et al., 2016). The virus is transmitted by the whitefly *Bemisia tabaci* (Gennadius) in a persistent manner.

The EOs and HSs antiphytoviral activity, foreseen in our hypothesis, was assessed by comparing the relative virus titer in systemically infected treated and untreated plants in order to ascertain if, and to what extent, treatments were able to decrease virus titer in a systemically infected leaf tissue of *C. pepo*. Several studies have established that plants were able to set up a defense response upon virus infection by using a wide range of regulation mechanisms (Soosaar et al., 2005). The phenylpropanoid pathway plays a role in such defense strategies by regulating the synthesis of phenolic compounds. Phenylalanine ammonia lyase (PAL) is the entry enzyme of this metabolic pathway, and the related gene is known to be involved in plant response to biotic stress through transcriptional regulation (Kim and Hwang, 2014; Abdelkhalek et al., 2018). Under the hypothesis of effectiveness of EOs and HSs treatments in plant virus control, PAL expression level was also quantified in treated and untreated infected plants in order to investigate the possible mechanism of action. Finally, symptom expression was also observed for evaluating disease incidence. To the best of our knowledge, this is the first report in which the EOs and HSs antiphytoviral activity is investigated *in vivo* on a vegetal species of agricultural interest and by means of quantification of viral titer and PAL expression levels by real-time (RT-)PCR.

## Materials and Methods

### Essential Oils and Hydrosols Production

Plants of OV, TV, and RO, which are used to produce EOs and HSs, were cultivated and harvested in Latium, central Italy at the agricultural experimental field Centro Appenninico del Terminillo “Carlo Jucci” in Rieti. EOs and HSs were produced by steam-distillation for 1 h using a Clevenger-type apparatus according to the procedures described in the *European Pharmacopeia*. Fresh leaves (2 kg) of aerial parts from each plant species were used for distillation. HSs were separated by decantation, and the EOs were treated twice with diethyl ether (Sigma-Aldrich, Italy) to eliminate the water through a separation funnel. EO/diethyl ether layer were dried with anhydrous sodium sulfate (Sigma-Aldrich, Italy) and the solvent was evaporated. EOs yields were 3.48, 0.26, and 0.43% for OV, TV, and RO, respectively. EOs were stored at −20°C and HSs at 4°C, both in the dark in glass vials.

### Gas Chromatography/Mass Spectrometry Analysis

Gas chromatography/mass spectrometry (GC-MS) analyses of EOs and HSs were performed using a Thermo Scientific gas chromatograph Focus GC, equipped with a fused silica capillary TG-SQC (30 m × 0.25 mm, film thickness 0.25 μm Thermo Fisher Scientific, United States) and a mass spectrometer 700 ITQ MS as detector. n-Hexane (97.0% grade) and all standard references (standard analytical grade) were supplied by Sigma Aldrich (Milan, Italy) with the exception of piperitenone oxide, which was in-house purified from *Mentha suaveolens* EO as previously described (Civitelli et al., 2014). Prior to injection, EOs were diluted 1:1,000 V/V with n-hexane, while HSs were previously liquid-liquid extracted in n-hexane 1:1 V/V and then diluted 1:100 V/V with n-hexane shortly before analysis. The organic fraction was separated, dried with anhydrous sodium sulfate, and the solvent was evaporated. GC-MS was performed according to the chromatographic and mass conditions described in Daferera et al. (2000), modified as follows: splitless mode injection, oven temperature program, 6 min of 60°C isothermal, then 3°C/min gradient up to 132°C, 10°C/min–180°C, and finally 15°C/min–240°C and held for 5 min. Injection volume was adjusted to 2 μl, while helium (99.9%) was the carrier gas at a constant flow rate of 1 ml/min. For GC-MS detection, detector temperature was set to 250°C, MS spectra were monitored between 50 and 450 amu (full scan mode), and the ionization mode used was an electronic impact at 70 eV. Compound identification was carried out to compare the relative retention times and mass spectra of the molecules with commercial libraries (NIST, 2017). When available, identification was confirmed by co-injection with a standard reference compound. Moreover, relative quantitation of these compounds was also accomplished by evaluating the relative percentage for each peak [peak area/total ion chromatogram (TIC) area] (Table 1).

**TABLE 1:** Phytochemical composition (%) of essential oils (EOs) and hydrosols (HSs) used in this study, by GC-MS.

Component	Rt (min)	OV EO	OV HS	TV EO	TV HS	RO EO	RO HS	Identification
***Monoterpene hydrocarbons***		*2.42*	*5.81*	*12.64*	*10.62*	*56.29*	*2.15*	
α-pinene	7.78	tr	−	0.22	−	32.55	1.94	MS
Camphene	8.40	−	−	tr	−	9.35	0.21	Co-GC
β-pinene	9.64	−	−	tr	−	2.06	−	Co-GC
Myrcene	10.40	−	−	0.18	−	2.31	−	Co-GC
Limonene	12.12	−	−	−	−	3.57	−	Co-GC
α-thujene	7.52	tr	−	tr	−	tr	−	MS
α-terpinolene	15.10	−	−	Tr	0.32	−	−	MS
α-terpinene	11.55	tr	−	0.23	−	0.55	−	Co-GC
γ-terpinene	13.64	−	5.04	0.60	−	0.84	−	Co-GC
p-cymene	11.93	2.27	0.77	11.78	10.30	5.03	−	Co-GC
***Oxygenated monoterpenes***		*5.45*	−	*6.05*	*27.35*	*26.49*	*95.55*	
Linalool	15.71	−	−	1.82	6.61	1.07	2.10	Co-GC
Camphor	17.69	0.71	−	0.40	1.90	6.67	20.89	Co-GC
Borneol	17.78	0.68	−	0.94	8.74	5.55	15.10	Co-GC
Terpinen-4-ol	19.36	0.92	−	2.06	6.47	0.42	1.62	Co-GC
Bornyl acetate	24.45	−	−	0.17	−	4.97	0.58	Co-GC
1,8-cineole	12.23	1.03	−	0.42	2.45	7.26	20.14	Co-GC
(Z)-p-menth-2-en-1-ol	16.66	−	−	tr	−	−	−	MS
α-terpineol	20.02	−	−	0.21	0.86	0.55	1.86	MS
p-cymen-8-ol	19.80	−	−	−	0.33	−	−	MS
Cis-sabinene hydrate	14.02	0.27	−	−	−	−	−	MS
Trans-sabinene hydrate	15.52	0.23	−	−	−	−	−	MS
Piperitenone oxide	28.01	1.62	−	−	−	−	−	MS
Verbenone	20.86	−	−	−	−	2.72	33.26	Co-GC
***Sesquiterpene hydrocarbons***		*0.53*	*1.06*	*2.35*	−	*5.24*	−	
β-caryophyllene	30.23	0.53	1.06	1.74	−	4.86	−	Co-GC
*Allo*-aromadendrene	32.70	−	−	Tr	−	tr	−	MS
Germacrene D	33.02	−	−	0.35	−	0.12	−	MS
β-copaene	32.10	−	−	0.17	−	tr	−	MS
α-cubebene	28.40	−	−	−	−	0.13	−	
***Oxygenated sesquiterpenes***		−	−	−	−	*tr*	−	
Caryophyllene oxide	34.90	−	−	−	−	tr	−	MS
***Phenolic compounds***		*90.62*	*93.13*	*75.95*	*61.04*	*1.83*	−	
Thymol	24.93	1.41	1.54	68.02	57.35	1.56	−	Co-GC
Carvacrol	25.34	88.64	91.59	5.71	3.69	tr	−	Co-GC
Thymol methyl ether	22.14	−	−	1.38	−	−	−	MS
Methyl carvacrol	22.56	0.57	−	0.83	−	0.16	−	MS
***Hydrocarbons***		*0.27*	−	*0.87*	−	−	−	
Cadina-1,3,5-triene	33.23	0.27	−	0.87	−	−	−	MS
***Alcohols***		−	−	*0.11*	*0.63*	−	−	
1-octen-2-ol	9.89	−	−	0.11	0.63	−	−	MS
Total identified		99.29	100.00	98.56	99.63	92.55	97.70	

*MS, identification by NIST (2017) spectral database; Co-GC, identification confirmed with reference compound; tr, traces (<0.1%). Values in bold represent the quantitation for the relevant class of compounds.*

### Plant Material

#### Plant Host

Seeds of *Cucurbita pepo* “Tullio” were sown in 12 cm plastic pots containing soil “Completo” (Vigorplant, Italy) and maintained in a greenhouse (23°C, 16 h light photoperiod) with watering as required. The obtained plants were grown in an insect-proof greenhouse under the same conditions. Experimental plants were selected 3 weeks after sowing, when they had two fully expanded cotyledons. Care was taken to ensure that the experimental plants were as uniform in size as possible.

#### Virus Inoculum

ZYMV and ToLCNDV isolates from the Research Centre for Plant Protection and Certification (CREA-DC) collection were propagated in the plant host *C. pepo* “Tullio” under the greenhouse conditions above described. Systemically infected young leaves were ground with chilled 0.1 M pH 7.4 phosphate buffer (1:5 w/V) in an extraction bag (Bioreba, Switzerland) to prepare virus *inocula* for the following experiments.

### Experimental Trials

#### Treatments Applied at the Same Time With Inoculation of Zucchini Yellow Mosaic Virus or Tomato Leaf Curl New Delhi Virus

The ZYMV or ToLCNDV *inoculum* was mixed with EO or HS solution in 0.1 M pH 7.4 phosphate buffer (final concentrations: 1:10 V/V virus *inoculum*; 300 μg/ml EO or 1:2 V/V hydrosol, see Table 2) and incubated for 1 h on ice. Then, the mixtures were used to mechanically inoculate host plants. For ZYMV trial, host plants were inoculated at the developmental stage of fully expanded cotyledons, and the 20 μl of mixtures ZYMV + treatment described above were applied to each cotyledon. For the ToLCNDV trial, plants were inoculated when they had cotyledons and the first true leaf fully expanded, and 80 μl were applied to each cotyledon and leaf. Three biological replicates were used per treatment, and controls were represented by (i) treatment with ribavirin (final concentrations: 1:10 V/V virus inoculum; 300 μg/ml ribavirin); (ii) infected no-treatment (final concentration: 10:10 V/V virus inoculum); and (iii) healthy (mock-inoculated with phosphate buffer). When the first new leaf was expanded 5 days post infection (d.p.i.), i.e., first true leaf for ZYMV trial, second true leaf for ToLCNDV trial, plants were sampled by removing a disk from the abovementioned leaf. Biological replicates (three plants per treatment) were pooled and downstream analyzed as a single sample. Each sample was thus submitted to quantification of virus titer and expression level of PAL gene by real-time (RT-)PCR as described below. The above described and following experimental procedures are depicted as a flow diagram in Supplementary Figure 1.

**TABLE 2:** Summary of treatments applied in experimental trials.

Entry	Product	Plant species	Concentration
1	EO	*Origanum vulgare*	300 μg/ml
2	HS	*Origanum vulgare*	1:2 V/V
3	EO	*Thymus vulgaris*	300 μg/ml
4	HS	*Thymus vulgaris*	1:2 V/V
5	EO	*Rosmarinus officinalis*	300 μg/ml
6	HS	*Rosmarinus officinalis*	1:2 V/V
7	Ribavirin	N/A	300 μg/ml

*Positive (i.e., infected no-treatment plants) and negative (i.e., mock-inoculated plants) controls were also included in each trial.*

#### Treatments Applied at the Same Time With Inoculation of Tomato Leaf Curl New Delhi Virus (Time Course)

Based on the results obtained for samples collected at 5 d.p.i. (see section “Treatments Applied at the Same Time With Inoculation of ZYMV or ToLCNDV”), the above trial on ToLCNDV was repeated, extending the period of observation, and after the first collection time point at 5 d.p.i., three more samplings were performed on a weekly basis. Each sampling involved a new leaf (i.e., third, fourth, and fifth leaf corresponding to sampling times 12, 19, and 26 d.p.i.), and as above, biological replicates were pooled and downstream analyzed as a single sample.

#### Treatments Applied After Inoculation of Tomato Leaf Curl New Delhi Virus (Time Course)

Based on the results obtained from the above-described trials, in which the treatments were applied concurrently with virus inoculation, further experiments were run only on ToLCNDV to evaluate the effect of the tested plant extracts post *inoculum*. In the literature, such activity is often addressed to as “curative” since the treated plants have already started the infection process. In this regard, host plants were inoculated in the same conditions described in section “Treatments Applied at the Same Time With Inoculation of ZYMV or ToLCNDV” (final concentration: 1:10 V/V virus *inoculum*), but no treatment was mixed with the *inoculum*. After 5 h, treatments were applied by smearing the leaf surface with the EO or HS solution described in section “Treatments Applied at the Same Time With Inoculation of ZYMV or ToLCNDV” (final concentration: 300 μg/ml EO or 1:2 V/V HS). Three biological replicates were used per treatment and controls were represented by (i) treatment with ribavirin (final concentration: 300 μg/ml); (ii) infected no-treatment (final concentration: 10:10 V/V virus inoculum); and (iii) healthy (mock-inoculated with phosphate buffer). When the first new leaf, i.e., the second true leaf, was expanded (5 d.p.i.), plants were sampled as above described. Biological replicates were pooled and downstream analyzed as a single sample. Additional samplings were performed as described in section “Treatments Applied at the Same Time With Inoculation of ToLCNDV (Time Course).”

### Nucleic Acid Extraction and Real-Time (RT-)PCR

Total RNA of samples collected from the ZYMV trial was extracted using RNeasy Plant Mini Kit (Qiagen, Milan, Italy). Total nucleic acid (TNA) of samples collected from the ToLCNDV trials (in treatments together with or after inoculation) was extracted using DNeasy “Mericon” Food Kit (Qiagen, Milan, Italy). Both kits were used according to the manufacturer’s instructions. Extracts were checked for purity and concentration by NanoDrop™ spectrophotometer (Thermo Fisher Scientific, Milan, Italy).

Total RNA samples (ZYMV trial) were treated with 2 U DNase I (Life Technologies, Milan, Italy) per 1 μg RNA, at room temperature for 15 min. Then, 1 μl of 50 mM EDTA was added, and the mix was incubated at 65°C for 10 min in a CFX96 Touch PCR System (Bio-Rad, Milan, Italy) to inactivate DNase and stop the reaction.

Total nucleic acid samples (ToLCNDV trial) were reverse transcribed to cDNA in order to convert plant transcriptome into DNA substrate suitable for real-time PCR. The reaction was carried out in a final volume of 19 μl using 5X first-strand buffer (Invitrogen), 5 μM random hexamers (Promega, San Diego, CA, United States), 10 μM dNTPs, 100 U M-MLV (Promega, San Diego, CA, United States), and 2 μl TNA. The reaction was incubated for 45 min at 42°C, 3 min at 94°C in a CFX96 Touch PCR System (Bio-Rad, Milan, Italy) before 1 μl RNase cocktail mix (Life Technologies, Milan, Italy) was added to remove residual unreacted RNA.

The relative quantification of ZYMV was carried out on total RNA, treated as described above with TaqMan^®^ real-time RT-PCR assay using the species-specific primers and probe designed on the coat protein (CP) gene by Manglli et al. (2019). The reaction had a final volume of 20 μl, containing 2X TaqMan^®^ RT-PCR Master Mix and 40X TaqMan^®^ RT Enzyme Mix (TaqMan^®^ RNA-to-C_*T*_™ 1-Step Kit, Life Technologies, Milan, Italy), 300 nM of each primer, 50 nM probe, and 1 μl DNase-treated RNA.

The relative quantification of ToLCNDV was carried out with TaqMan^®^ real-time PCR assay using the species-specific primers and probe designed on the A genome of ToLCNDV by Simón et al. (2018). The reaction had a final volume of 10 μl containing 2X TaqMan^®^ RT-PCR Master Mix, 100 nM of each primer and probe, and 1 μl of RNase-treated cDNA.

The relative expression of PAL was quantified with SYBR Green^®^ real-time RT-PCR assay using primers designed by Zhang et al. (2021). The reaction had a final volume of 10 μl containing 2X SsoAdvanced Universal SYBR Green Supermix (Bio-Rad, Milan, Italy), 150 nM of each primer, and 1 μl of template.

All real-time (RT-)PCR assays were performed on a CFX96 Touch RT-PCR System (Bio-Rad, Milan, Italy). The threshold was automatically set by the instrument.

The virus relative titer and PAL expression level were determined using the method of ΔΔCt (Livak and Schmittgen, 2001). The elongation factor EF-1α gene of *C. pepo* was used as housekeeping for both assays using previously published primers and probe (Obrero et al., 2011). All samples represented a pool of the three biological replicates (i.e., plants in the same pot) and were assayed in two technical replicates. Relative virus titer and PAL expression level, defined as 2^–ΔΔCt^ were calculated by CFX Maestro Software v. 2.2 (Bio-Rad, Milan, Italy) and the results were expressed as mean ± standard error (SE).

## Results

Plant extracts (EOs and HSs) from OV, TV, and RO were characterized by GC-MS in order to assess their specific composition, which is known to strongly depend on environmental and plant characteristics.

**FIGURE 1:**
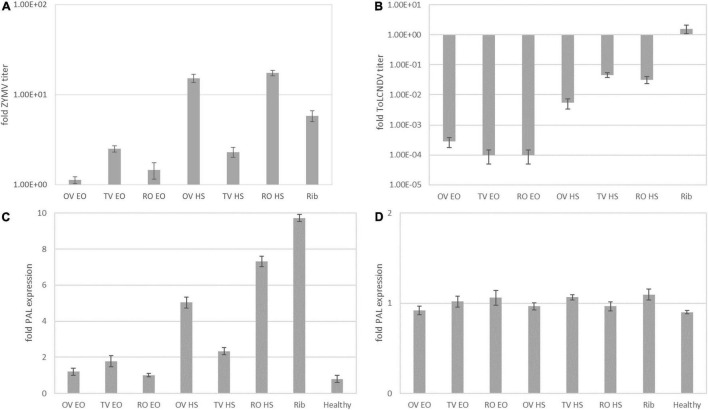
Fold change of zucchini yellow mosaic virus coat protein (ZYMV-CP) **(A)**, tomato leaf curl New Delhi virus (ToLCNDV)-A **(B)**, and relative expression of phenylalanine ammonia lyase (PAL) in ZYMV-infected treated **(C)** and ToLCNDV-infected treated **(D)** at 5 d.p.i. with respect to infected no-treatment control. OV EO, *O. vulgare* essential oil; TV EO, *T. vulgaris* essential oil; RO EO, *R. officinalis* essential oil; OV HS, *O. vulgare* hydrosol; TV HS, *T. vulgaris* hydrosol; RO HS, *R. officinalis* hydrosol; Rib, ribavirin; Healthy, mock-inoculated plants. Columns represent mean value from 2 technical replicates on 3 pooled biological replicates, and bars indicate standard error (± SE).

**FIGURE 2:**
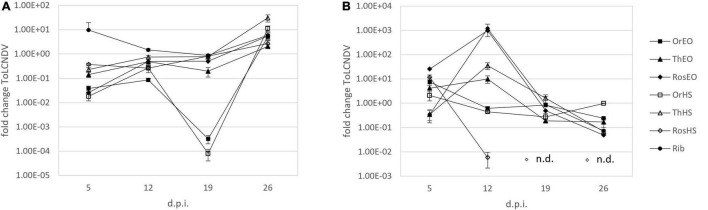
Fold change of ToLCNDV-A in plants treated together with inoculation **(A)** and treated after inoculation **(B)** of ToLCNDV on leaves harvested at 5, 12, 19, and 26 d.p.i. OV EO, *O. vulgare* essential oil; TV EO, *T. vulgaris* essential oil; RO EO, *R. officinalis* essential oil; OV HS, *O. vulgare* hydrosol; TV HS, *T. vulgaris* hydrosol; RO HS, *R. officinalis* hydrosol; Rib ribavirin. Columns represent mean value from 2 technical replicates on 3 pooled biological replicates and bars indicate standard error (± SE).

Under greenhouse conditions, the antiviral activities of these plant extracts were evaluated on *C. pepo* plants experimentally inoculated with either ZYMV or ToLCNDV. Using real-time PCR, both the relative virus titer and the relative expression of PAL were measured on treated plants with respect to control plants (i.e., infected no-treatment). Symptoms were also recorded on the inoculated test plants in order to observe possible phenotypical modification and symptom intensity and variability induced by treatments. Preliminarily, symptoms on control plants were evaluated and resulted as expected. In all experiments, the inoculated no-treatment control plants displayed symptoms of the relevant infection. Healthy no-treatment control plants coherently showed no symptoms at any time. They did not display any signal in real-time PCR assays for virus presence and were not included in relevant figures (Figures 1A,B, 2).

### Gas Chromatography/Mass Spectrometry Analysis

The compounds identified in EOs and HSs by GC-MS are listed in Table 1. Thirty-four compounds belonging to 7 chemical classes and 22 compounds belonging to 5 classes were identified in EOs and HSs, respectively. In all cases, identified compounds totally accounted for 92.55–100% of EO or HS. Phenolic compounds, mostly thymol and carvacrol, were main constituents of EOs and HSs from OV and TV, while RO EO and HS were largely composed of monoterpene hydrocarbons and oxygenated monoterpenes, respectively.

In OV EO, carvacrol accounted for 88.64% composition, followed by minor amounts of p-cymene (2.27%), thymol (1.41%), and piperitenone oxide (1.62%). As expected, due to the polarity of its phenolic moiety, a large amount of carvacrol (91.95%) was also found in the respective HS. Thymol was also present in HS and EO in similar amounts. Thymol was the main constituent of TV EO (68.02%), but in this case, p-cymene and carvacrol were also found in fairly high proportions (11.78 and 5.71%, respectively). As in the case of OV extracts, the main component of TV HS was the same of the corresponding EO (57.35%) and compounds bearing oxygenated polar groups as oxygenated monoterpenes and phenolic compounds were found in similar or higher proportions (borneol 8.74%, linalool 6.61%, terpinen-4-ol 6.47%, carvacrol 3.69%). The highest number of identified compounds (26) characterized RO EO. Its composition was described in terms of a main constituent α-pinene, accounting for 32.55% composition, followed by 7 compounds at medium concentrations ranging from 4.86 to 9.35% that belong to monoterpene hydrocarbons, oxygenated monoterpenes, and sesquiterpene hydrocarbon classes, and 13 minor components (0.12–3.57%). In the corresponding HS, oxygenated monoterpenes were main components (verbenone 33.26%, camphor 20.92%, 1,8 cineole 20.72%, borneol 15,12%).

### Virus Quantification and Symptoms Expression

The relative virus titer was determined by the ΔΔCt method based on the Ct values obtained by real-time PCR amplifications and considering the EF-1α gene as the housekeeping gene and the infected no-treatment group as the control.

#### Treatments Applied at the Same Time With Inoculation of Zucchini Yellow Mosaic Virus or Tomato Leaf Curl New Delhi Virus

**FIGURE 3:**
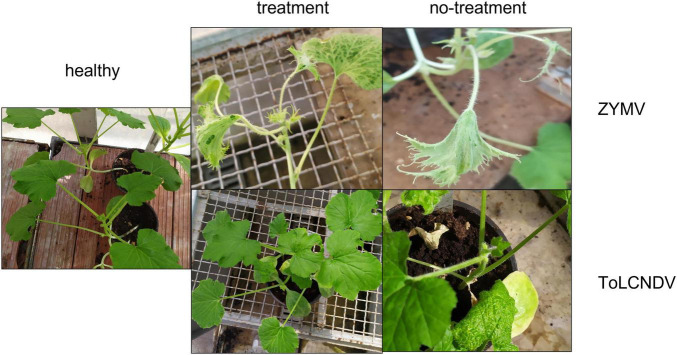
Visual inspection of plants treated together with inoculation of ZYMV or ToLCNDV: one example treatment and the relevant no-treatment is shown for each virus.

Figure 1 reports the fold change of relative virus titer (i.e., 2^–ΔΔCt^) for the experimental trial vs. ZYMV and ToLCNDV described in section “Treatments Applied at the Same Time With Inoculation of ZYMV or ToLCNDV.” The ZYMV relative virus titer (Figure 1A) increased with respect to the infected no-treatment control in all treatments. Hence, in treated plants, a stimulation of virus replication rather than inhibition was observed. This effect was particularly prominent in OV and RO HS treatments. Also, the ribavirin-treated positive control gave an increase of relative virus titer. The observation of symptoms also confirmed a noticeable virus infection on all treated plants, with severe malformation of new and systemically infected leaves (Figure 3).

Results on ToLCNDV are reported in Figure 1B. The decrease of relative ToLCNDV titer with respect to infected no-treatment control was recorded in all treatments, with fold changes ranging from 10^–3^ to 10^–4^ for EOs and from 10^–2^ to 10^–1^ for HSs.

In both trials, ribavirin did not show to work as a proper positive treatment control, giving rise to an increase of relative ZYMV titer and being quite ineffective on the relative ToLCNDV titer, with respect to infected no-treatment controls.

The visual inspection of the test plants recorded severe symptoms (mosaic, malformation, blistering, stunting on leaves) on ZYMV-infected plants, independently of the treatment type. Conversely, only mild yellow mosaic was observed on treated plants inoculated with ToLCNDV, whereas severe symptoms (i.e., leaf curling, vein swelling, plant stunting) were only observed in the infected no-treatment control (Figure 3).

Based on these results, further experiments were performed on ToLCNDV, which proved to be more affected by EOs and HSs treatments than ZYMV, both in virus titer decrease and symptom development.

#### Treatments Applied at the Same Time With Inoculation of Tomato Leaf Curl New Delhi Virus (Time Course)

**FIGURE 4:**
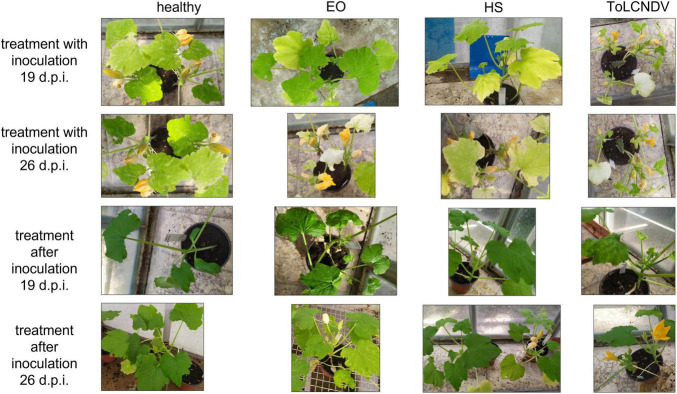
Visual inspection of plants treated together with inoculation (upper panels) and after inoculation (lower panels) at 19 and 26 d.p.i.; the relevant healthy and infected no-treatment control is shown for each entry.

The evolution of ToLCNDV relative titer in leaves of treated *C. pepo* plants over time was investigated by means of an extended trial involving repeated weekly samplings until 26 d.p.i. as described in section “Treatments Applied at the Same Time With Inoculation of ToLCNDV (Time Course).” Figure 2A shows the fold change values obtained for relative ToLCNDV titer at different sampling times for all treatments. In the first sampling (i.e., 5 d.p.i.), the results were quite similar to the previous experiment on ToLCNDV (section “Treatments Applied at the Same Time With Inoculation of ZYMV or ToLCNDV”). At 12 d.p.i., the relative titer increased with respect to the first sampling in nearly all treatments (RO HS remaining about the same), but it remained lower than the infected no-treatment control, with fold changes ranging from 10^–2^ to 10^–1^. At 19 d.p.i., treatments with either OV EO or OV HS were still very effective in reducing virus titer (i.e., 10^–4^–10^–3^-fold change). Virus titer in other treatments (i.e., TV and RO EOs and HSs) was comparable to that measured at 12 d.p.i. In the final sampling, i.e., 26 d.p.i., an increasing of virus titer ranging from 10^1^ to 10^2^ -fold was recorded in all treatments with respect to no-treatment infected control. EOs generally recorded increases of lower extent than HSs. Ribavirin treatment confirmed not to be a suitable positive control since an increase of virus titer was observed at all sampling times. The evolution of symptoms expression during time is shown in Figure 4, upper panels, in which at both 19 and 26 d.p.i., EO treated plants were displaying less symptoms with respect to HS plants, and more similar to healthy control.

#### Treatments Applied After Inoculation of Tomato Leaf Curl New Delhi Virus (Time Course)

The experimental trial described in section “Treatments Applied After Inoculation of ToLCNDV (Time Course)” was designed to evaluate the effectiveness of treatments when applied after virus inoculation (i.e., curative effect). In this case, at 5 d.p.i., ToLCNDV titer was higher in all treatments with respect to no-treatment infected control, except for TV HS. Here, we observed a viral titer reduced to one-third of the control (Figure 2B). In the sampling at 12 d.p.i., three treatments (i.e., OV EO and HS, and RO HS) showed a decrease of ToLCNDV titer compared to the control. Particularly, results were ca. 10^–1^-fold decrease for OV EO and HS and ca. 10^–3^-fold decrease for RO HS. At 19 d.p.i. in all treatments except TV HS, a decrease of virus titer was observed. In RO HS, as in the previous sampling, the decrease was most remarkable, with the virus target not detected at all in the samples. In the final sampling at 26 d.p.i., all treatments showed a virus titer lower than the control: fold changes ranged from 10^–1^ for OV EO and HS and TV EO to 10^–2^ for RO EO and TV HS. The most prominent effect was again observed in RO HS treatment, in which the virus was not detected. Pictures of the plants of these experiments at 19 and 26 d.p.i are shown in Figure 4, lower panels, in which HS plants appeared less diseased than EO plants, with the latter displaying leaf mosaic and curling. This observation is in agreement with the results of virus titer quantification. Specifically, HS plants shown in the pictures were those treated with RO HS, appearing completely free from disease symptoms and identical to healthy control, both at 19 and 26 d.p.i. In the same plants, ToLCNDV was not detected by real-time PCR at the same d.p.i.

### Phenylalanine Ammonia Lyase Gene Expression

The relative PAL expression was determined by the ΔΔCt method based on the Ct values obtained by real-time PCR amplifications and considering the EF-1α gene as the housekeeping gene and the infected no-treatment group as the control. In this work, PAL represents the first gene analyzed to investigate the antiviral mode of action performed by EOs or HSs in plant defense response.

#### Treatments Applied at the Same Time With Inoculation of Zucchini Yellow Mosaic Virus or Tomato Leaf Curl New Delhi Virus

Phenylalanine ammonia lyase gene relative expression levels were measured at 5 d.p.i. on treated plants compared to the infected no-treatment control for the two experimental trials on ZYMV and ToLCNDV described in section “Treatments Applied at the Same Time With Inoculation of ZYMV or ToLCNDV.” The transcription levels of PAL were significantly upregulated in treated leaves infected with ZYMV (Figure 1C). In HS treatments, the relative expression levels increased by 5.0, 2.3, and 7.3-fold compared to the control for OV, TV, and RO, respectively. Such performance of HS treatment was of the same order of magnitude of ribavirin treatment (9.7-fold increased PAL expression level).

In ToLCNDV-infected plants (Figure 1D) no significant increase in PAL expression levels associated to any treatment was recorded. In order to better investigate and confirm or discard this trend, a time course experiment was run with a higher number of samplings as described in section “Treatments Applied at the Same Time With Inoculation of ToLCNDV (Time Course)”.

#### Treatments Applied at the Same Time With Inoculation of Tomato Leaf Curl New Delhi Virus (Time Course)

**FIGURE 5:**
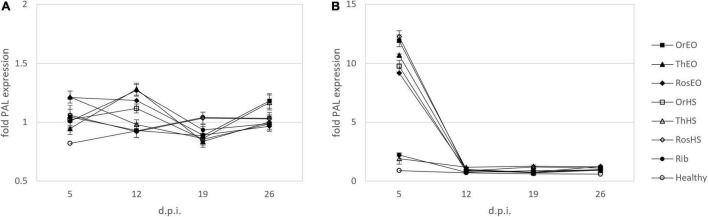
Relative expression of PAL in plants treated together with inoculation **(A)** and treated after inoculation of ToLCNDV **(B)** on leaves harvested at 5, 12, 19, and 26 d.p.i. OV EO, *O. vulgare* essential oil; TV EO, *T. vulgaris* essential oil; RO EO, *R. officinalis* essential oil; OV HS, *O. vulgare* hydrosol; TV HS, *T. vulgaris* hydrosol; RO HS, *R. officinalis* hydrosol; Rib, ribavirin; Healthy, mock-inoculated plants. Columns represent mean value from 2 technical replicates on 3 pooled biological replicates and bars indicate standard error (± SE).

The evolution of PAL expression over time in all treatments when applied at the same time of inoculation is shown in Figure 5A. An increased level of PAL transcript was recorded in treatments with TV and RO EOs at 12 d.p.i. (i.e., 1.3 and 1.2-fold higher than infected no-treatment control, respectively, and similar to ribavirin treatment). In samples collected at later times, no significant difference was evidenced between treatments and control.

#### Treatments Applied After Inoculation of Tomato Leaf Curl New Delhi Virus (Time Course)

Phenylalanine ammonia lyase gene expression levels were also studied during the time when treatments were applied 5 h after inoculation (curative effect, Figure 5B). In the first sampling, at 5 days d.p.i., PAL transcript showed the greatest increase observed in our experiments, with all treatments except TV HS reaching the order of magnitude of 10-fold increase with respect to control. This trend was weaker in the following observations: treatments showing signs of upregulation (i.e., TV HS at 12, 19, and 26 d.p.i, RO HS at 19 and 26 d.p.i, RO EO at 26 d.p.i.) had relative expression levels of ca. 1.2. Other treatments did not record any change in the expression of PAL with respect to infected no-treatment control.

## Discussion

The use of natural products from plant sources for the treatment of plant viruses is a topic of growing interest and application. Previous studies demonstrated that EOs and HSs are effective in reducing the number of local lesions due to virus infection in experimental hosts for a number of plant/virus pathosystems (Bishop, 1995; Dunkić et al., 2010; Bezić et al., 2011; Lu, 2013; Vuko et al., 2019, 2021a,b; Nazlić et al., 2021). Furthermore, gene expression studies were performed in order to identify genes and metabolic pathways involved in this action (Vuko et al., 2019). In this study, we aimed at broadening the state-of-the-art knowledge by testing two typologies of natural products (i.e., EOs and HSs) *in vivo* on *C. pepo* plants infected by two viruses. The choice of the plant species sources of extracts to be tested was based on preliminary results from a screening of 60 EOs from different officinal plant species, indicating that OV, TV, and RO EOs can significantly decrease (Student’s *t*-test) the number of local lesions in *Chenopodium amaranticolor* infected by ZYMV (data not shown, manuscript in preparation). The respective HSs from the same plant species were also included in experiments in view of the recent literature supporting biological activity of HSs as antimicrobials and antivirals (D’Amato et al., 2018; Vuko et al., 2021a,b). *C. pepo* was chosen as the host plant, being an important agricultural crop and not a model experimental host but sharing the features of easy management in greenhouse raising, artificial inoculations, and setting up of experimental trials involving biological replicates. The target viruses (i.e., ZYMV and ToLCNDV) were selected considering the great impact they have on *C. pepo* crops, causing diseases responsible for significant economic losses. Moreover, the two viruses are considerably different in some features which could be relevant for the liability of treatments, i.e., symptomatology, taxonomy, geographical distribution, genome structure and organization, and transmission mode. Resistant varieties are currently used for the control of ZYMV in *C. pepo*, but resistance breaking (RB) viral strains are becoming more and more frequent. For ToLCNDV, being an emerging pathogen, no direct control measure is yet available. Hence, for both viruses, alternative control strategies are needed. In this study, we hypothesized that the above mentioned EOs and HSs exert an antiphytoviral activity against ZYMV and ToLCNDV in *C. pepo*. We aim at demonstrating the effectiveness of such treatments in terms of virus titer and symptoms development. Further, we explore a possible mechanism of action by the quantification of PAL expression levels. These findings provide quantitative data on the effectiveness of EOs and HSs in virus control, improving the knowledge on this important topic. Our data could represent a basis for future scientific research on the mechanism of action of such treatments, and the formulation of a possible remedy which is needed in *C. pepo* cropping.

The qualitative and quantitative EO compositions reported in this work are overall in fair agreement with literature data, considering the intrinsic variability of these extracts due to a number of factors influencing the biosynthesis and metabolism of EOs components. Exogenous factors (i.e., temperature, photoperiod, soil, agronomic conditions, harvesting season, distillation methodology, plant chemotype, and cultivar) and endogenous features (i.e., part, tissue, age of the plant) are all aspects reported to strongly affect EOs composition (Barra, 2009). In OV EO, we identified carvacrol as main compound, with minor amounts of p-cymene and thymol as confirmed by previous works (Béjaoui et al., 2013; Soltani et al., 2021). The prevalence of thymol in TV EO used in our experiments identified it as belonging to the thymol chemotype (Thompson et al., 2003). RO EO was characterized by a broader composition whose main components (i.e., α-pinene, camphene, 1,8 cineole) were all reported in the literature as identified in various geographical origin RO extracts (Verma et al., 2020). HSs are still poorly studied with respect to EOs. Hence, limited data can be found in the literature regarding their main components. The composition of some HSs closely resembles that of their related EOs. Nevertheless, in some cases, HSs were reported to be completely different in quantitative and qualitative terms (Inouye et al., 2008). A general trend has risen from the analysis of several HSs compared with the associated EOs, indicating oxygenated compounds (i.e., aldehydes, alcohols, phenols, ketones, esters, and phenol methyl ethers) to often be main components (Inouye et al., 2008), while monoterpene and sesquiterpene hydrocarbons, though present in high concentrations in EOs, were quite absent in most HSs (Bellahsene et al., 2015). In agreement with this outline and with previous literature (Khan et al., 2018), carvacrol was the main compound of OV HS in our study, accounting for more than 90% of total composition. In agreement with literature data, thymol was the main component of TV HS (D’Amato et al., 2018) and in the corresponding EO. RO HS in our experiments was characterized by a broader composition, as was also established for its related EO. Particularly, none of the components exceeded 33% of the total. Four oxygenated monoterpenes (verbenone, camphor, 1,8 cineole, and borneol), which were constituents of EO, were found in rather high proportions in HS.

Experiments in which the treatment was simultaneously applied with virus inoculation indicated an effective antiphytoviral activity vs. ToLCNDV, especially for treatments with EOs. This can probably be attributed to phenolic main compounds in OV and TV EOs, and thymol and carvacrol, having been reported as antiphytovirals (Dunkić et al., 2010). Due to the more complex composition of RO EO, speculations on the components most likely playing such biological activity are not possible. Hence, further investigation is needed. Conversely, no significant virus titer decrease was observed in ZYMV infected plants. In addition, treatments with OV and RO HSs resulted in more than 10-fold increased ZYMV titer. Further, whereas the visual inspection of symptoms provided evidence of an actual inhibition of ToLCNDV, an increase of disease severity in ZYMV infected treated plants was recorded. On the contrary, inhibition effect on ZYMV was observed in preliminary experiments on *C. amaranticolor* as described above. We hypothesize that such huge difference in activity by the same treatments against different plant or virus species could be related to the mechanism of action and nature of either the host or the virus. The poor results on ZYMV were, however, in agreement with previous literature. Particularly, that a number of EOs, including RO EO, did not display any activity against TMV *in vitro* at concentration of the same order of magnitude of our study (Lu, 2013).

For the different effectiveness against ZYMV infection on the two plant hosts, it has to be emphasized that local infection was evaluated in *C. amaranticolor*, while in experiments on *C. pepo*, systemic infection on new leaves was assayed. Hence, in the case of ZYMV, treatments could be effective locally but not systemically. In this context, the increased PAL expression levels recorded in ZYMV-infected treated plants was probably not meaningful. PAL may not be involved in effective defense response to ZYMV infection in our experimental conditions. To date, the increase of PAL expression levels as a response to virus control strategies has only been reported in case of resistance/tolerance mechanisms (Kavil et al., 2021).

A notable difference in activity was also recorded on the same host, against the two viruses, and both assayed in systemic infection. ToLCNDV and ZYMV have been classified apart in the largest subdivision of plant viruses, which was based on the chemical structure of their genomes: ToLCNDV is a ssDNA virus, while ZYMV has an RNA genome. This aspect could represent a possible reason for the different activity of extracts. Due to the unlike intrinsic nature of the genetic material, their life cycles are often very distinct from each other. For example, in the infection process, DNA viruses require a transcription step, and must be translocated to the nucleus for such stage, whereas both types need a translation step (Gergerich and Dolja, 2006). The reported and well-known activity of EOs and HSs against pathogens with DNA genome, such as bacteria and fungi (Bakkali et al., 2008; D’Amato et al., 2018), was found in agreement with the hypothesized target pest genome chemical structure role in the control of pathogenesis exerted by plant extracts. This could make DNA viruses generally more susceptible than RNA viruses to such treatments.

The positive control included in the trial (Table 2) was represented by treatment with ribavirin, a broad-spectrum antiviral guanosine analog (Sidwell et al., 1972). Nucleoside analogs, bearing a modified nitrogenous base, exert antiviral activity by converting into nucleotide metabolites, causing mutations in viral RNA and thus increasing the frequency of defective viral RNA. Ribavirin is reported to have inhibitory activity on plant viruses (Cassells, 1983; Lerch, 1987) and is currently used as a chemotherapy agent for *in vitro* virus elimination on many plant species (Panattoni et al., 2013; Wang et al., 2018). It had been included as a synthetic benchmark for evaluating antiphytoviral activity. Due to the unavailability of actual compounds active against plant viruses in agronomic practices, any comparison with a well characterized remedy was prevented to set up. Despite this, in our experiments, it is notable that ribavirin control did not work as expected.

The extended trial of ToLCNDV-infected treated plants was set up based on the promising data arising from the first experiment involving one single sampling at 5 d.p.i. In fact, further analyses covered a period until 26 d.p.i. and let us gain more information on the evolution of infection. In particular, when treatments were applied with inoculation, the virus titer was still lower in treated than untreated plants at 19 d.p.i. and only started to exceed the control values at 26 d.p.i. At that time, it was also noticeable that EO treatments generally caused a milder increase of virus titer with respect to HSs. We can therefore conclude that, in these experimental conditions, EOs’ activity was generally slightly longer-lasting than HSs’. This could be due to the differences in amounts of active compounds and/or in chemical nature (i.e., EOs hydrophobic, HSs hydrophilic) of the two types of extract. Hence, this could result in diverse viability of treatments on plant tissues.

Virus infection evolution and plant response were also investigated in treatments applied after inoculation. The effects of treatments were generally delayed but longer lasting compared to treatments simultaneously applied with virus *inoculum*. An effective decrease of virus titer was observed only after the second sampling time, regardless of treatments. Particular attention should be paid to the remarkable effect of RO HS. Particularly, in plants treated with such extract, the virus was not detected by real-time PCR at 19 and 26 d.p.i. Additionally, tested plants did not show any symptom. The results of such time-course observations suggested that the treatments’ effect on symptoms was probably longer lasting than previous studies reported. In literature, the observation was limited to one single time point, to the few hours immediately after treatment, or within a maximum period of 7 days (Helal, 2019; Vuko et al., 2021b).

Besides the assessment of virus titer, the quantification of PAL expression levels was also accomplished in order to verify whether this gene is involved in the plant defense response stimulated by treatments. PAL is the entry enzyme of the phenylpropanoid pathway, and phenylpropanoid compounds are well known as key elements in plant defense against many microbial pathogens (La Camera et al., 2004). Hence, PAL is one of the most extensively studied enzymes in the field of plant response to biotic and abiotic stress. It has also been reported to be upregulated in case of virus infection (Gutha et al., 2010; Abdelkhalek et al., 2020) and in the set-up of resistance development (Kim and Hwang, 2014; Abdelkhalek et al., 2018) in various plant/pathogen systems.

When treatments were simultaneously applied with virus *inoculum*, this quantification showed that TV extracts were effective in increasing accumulation of PAL transcripts, especially at 12 d.p.i. TV EO has recently been reported to trigger such effect. For this elicitor activity, it has been proposed for sustainable plant protection against pathogens (Bill et al., 2017). Our results showed an early response of PAL to biotic stress that is limited to 5–12 d.p.i. In this context, earlier sampling times (i.e., < 5 d.p.i.) were envisaged in order to explore the trend of PAL transcripts in the very first few hours/days after treatment. This strategy was actually limited by the requirement to systemically sample infected new leaves. Hence, a period of delay between treatment and first sampling, which was necessary for the development of the first new, not locally inoculated/treated leaf, appeared to be unavoidable.

In the assessment of curative activity, we recorded the highest response in terms of PAL transcripts in all treatments except TV HS. The effect was limited to 5 d.p.i., while following observation points led to transcript levels similar to the control. In view of the above, we speculated that at least one part of the defense response mechanism triggered by EOs and HSs against ToLCNDV involved a modulation of expression of PAL gene.

## Conclusion

Carvacrol and thymol were the main constituents of EO and HS extracted from OV and TV, while RO extracts displayed a more complex chemical composition, mostly monoterpene hydrocarbons in EO and oxygenated monoterpenes in HS. Antiphytoviral assays showed a decrease of ToLCNDV titer, while no or adverse effects of treatments were recorded against ZYMV. The application of treatments after inoculation, rather than simultaneously, led to a longer-lasting effect on the virus titer. In such conditions, the level of PAL transcripts also recorded a more prominent increase than in the co-inoculation of virus and treatment, although limited to the first sampling time. In our experiments, the best performance combination of treatment, mode of application, and virus was displayed by RO HS applied after ToLCNDV inoculation. In such conditions, the virus was not detected on systemically infected leaves by real-time PCR at 19 and 26 d.p.i.

Overall, our results support the feasibility of using OV, TV, and RO EOs and HSs as potential treatments for ToLCNDV-infected *C. pepo* plants under greenhouse conditions. Further investigation is needed to evaluate the applicability of such treatments for practical use in greenhouse and in the field.

## Data Availability Statement

The raw data supporting the conclusions of this article will be made available by the authors, without undue reservation.

## Author Contributions

AT and LT: conceptualization. AT, LT, and RR: methodology. AT, LD, and PN: formal analysis and data curation. AT, LD, FS, MS, GD, SF, VV, and RR: investigation. AT: writing—original draft preparation and visualization. AT, LD, LF, LT, MS, FS, and RR: writing—review and editing. AT and RR: supervision. All authors have read and agreed to the published version of the manuscript.

## Conflict of Interest

The authors declare that the research was conducted in the absence of any commercial or financial relationships that could be construed as a potential conflict of interest.

## Publisher’s Note

All claims expressed in this article are solely those of the authors and do not necessarily represent those of their affiliated organizations, or those of the publisher, the editors and the reviewers. Any product that may be evaluated in this article, or claim that may be made by its manufacturer, is not guaranteed or endorsed by the publisher.

## Supplementary Material

Supplementary Figure 1 Flow diagram of the experimental procedures described in section “Treatments Applied at the Same Time With Inoculation of ZYMV or ToLCNDV”—“Treatments Applied at the Same Time With Inoculation of ToLCNDV (Time Course)” (flow **A**), and in section “Treatments Applied After Inoculation of ToLCNDV (Time Course)” (flow **B**).
