# Supplementary figures and images for: In vivo Antiphytoviral Activity of Essential Oils and Hydrosols From Origanum vulgare, Thymus vulgaris, and Rosmarinus officinalis to Control Zucchini Yellow Mosaic Virus and Tomato Leaf Curl New Delhi Virus in Cucurbita pepo L
Source: Front Microbiol. 2022 Apr 25;13:840893. doi: 10.3389/fmicb.2022.840893 (PMC9085358; doi:10.3389/fmicb.2022.840893)

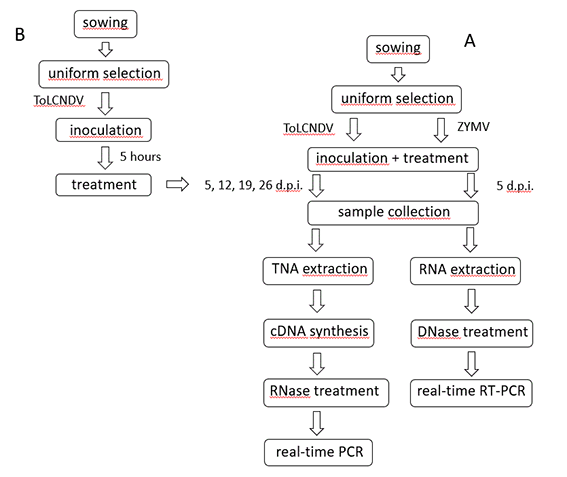

Supplement: Supplementary Figure 1 — Flow diagram of the experimental procedures described in section “Treatments Applied at the Same Time With Inoculation of ZYMV or ToLCNDV”—“Treatments Applied at the Same Time With Inoculation of ToLCNDV (Time Course)” (flow A), and in section “Treatments Applied After Inoculation of ToLCNDV (Time Course)” (flow B). [file Image_1.TIFF]
